# The effect of gradually lifting the two-child policy on demographic changes in China

**DOI:** 10.1093/heapol/czae008

**Published:** 2024-02-09

**Authors:** Yidie Lin, Baiyang Zhang, Meijing Hu, Qiang Yao, Min Jiang, Cairong Zhu

**Affiliations:** Department of Epidemiology and Health Statistics, West China School of Public Health and West China Fourth Hospital, Sichuan University, No. 16 People’s South Road, Chengdu 610041, China; Department of Epidemiology and Health Statistics, West China School of Public Health and West China Fourth Hospital, Sichuan University, No. 16 People’s South Road, Chengdu 610041, China; Department of Epidemiology and Health Statistics, West China School of Public Health and West China Fourth Hospital, Sichuan University, No. 16 People’s South Road, Chengdu 610041, China; Department of Epidemiology and Health Statistics, West China School of Public Health and West China Fourth Hospital, Sichuan University, No. 16 People’s South Road, Chengdu 610041, China; Department of Epidemiology and Health Statistics, West China School of Public Health and West China Fourth Hospital, Sichuan University, No. 16 People’s South Road, Chengdu 610041, China; Department of Epidemiology and Health Statistics, West China School of Public Health and West China Fourth Hospital, Sichuan University, No. 16 People’s South Road, Chengdu 610041, China

**Keywords:** Birth policy, birth rate, population growth, synthetic control

## Abstract

Low-fertility rate has been a common problem in many industrialized countries. To reverse the declining trend of new births, Chinese government gradually lifted its restrictions on the number of births per family, allowing for a household to have no more than two children. Little is known about the additional births or population increase contributed by the gradual relaxation of birth restrictions. To fill this gap, this quasi-experimental design study including data from 124 regions used the synthetic control method and controlled interrupted time series analysis to evaluate the differences in birth rates and rates of natural population increase between China and its synthetic control following implementation of the two-child policy from 2011 to 2020. A total of 123 regions were included in the control pool. Data collected during 1990–2010 were used to identify the synthetic China for each study outcome. The mean rate differences of birth rates and rates of natural increase between China and synthetic China after two-child policy implementation were 1.16 per 1000 population and 1.02 per 1000, respectively. These rate differences were distinguished from variation due to chance (one-sided pseudo-*P*-values: *P* for birth rates = 0.047, *P* for rates of natural increase = 0.020). However, there were statistically significant annual reductions in the pre–post trend of birth rates and rates of natural increase compared with those of controls of <0.340 per 1000 population per year [*P *= 0.007, 95% CI = (−0.584, −0.096)] and <0.274 per 1000 per year [*P *= 0.028, 95% CI = (−0.518, −0.031)]. The results suggested that lifting birth restrictions had a short-term effect on the increase in birth rates and rates of natural population increase. However, birth policy with lifting birth restrictions alone may not have sustained impact on population growth in the long run.

Key messages **1. Implications for policymakers**

Lifting birth restrictions had a short-term effect on the increase in birth rates. However, the shift from a birth control policy to lifting birth restrictions has no sustained impact on population increase in the long run. Birth restrictions seem unnecessary in contemporary China and the government should make attempts to construct various financial supportive systems as quickly as possible so as to encourage more births of a family.

**2. Implications for public**

The results of this study support the calling for cancelling any birth restrictions on the number of births per household. Moreover, the deep reasons for the rapid declining of birth rates should be investigated to support couples at childbearing age to give more births.

## Introduction

Birth rates are declining in many industrialized countries and have been doing so for >30 years, which brings about the challenges of labour force shortage, population ageing and pension burden. In response to the decline in birth rates, China’s policymakers performed a transition from the one-child policy to the two-child policy (TCP). The one-child policy, initiated in 1979, aimed to address overpopulation, severe poverty and resource scarcity issues (Hesketh and Zhu, 1997). The one-child rule was strictly enforced for urban residents but was less popular and deemed virtually unenforceable in rural areas. From 1984 onwards, rural couples in some provinces were allowed a second child if the firstborn was female (Zhang, 2017). However, over the past 40 years, rapid socioeconomic development and a declining fertility rate have led to an ageing population and workforce shortages (Zeng and Hesketh, 2016). These issues have raised public awareness and accelerated the process of lifting birth restrictions. The first phase of the selective two-child policy (STCPI) was announced in 2011, allowing couples who were both singletons to have two children. The second phase (STCPII), from 2013 to 2015, extended the standard to couples in which at least one of the marital partners was an only child. However, by May 2015, only 13.2% of 11 million eligible couples had applied to have a second child. This low uptake, along with appeals from scholars and the media, probably accelerated the announcement of the universal two-child policy (UTCP). In October 2015, the government declared that all couples in China’s mainland could have two children, which marked the official end of the one-child policy.

In fact, China is not the only country that has experienced a birth policy shift. For example, Iranian policymakers shifted away from a birth control policy towards a pronatalist policy in 2012, yet the trend of slowing population growth has not been reversed after lifting birth restrictions (Erfani, 2017; Bagheri *et al*., 2021). Similarly, in typical low-fertility countries, pronatalist policies with various financial support strategies have not always succeeded in increasing birth rates (Gauthier, 2002; McDonald, 2006). Thus, the main controversy surrounding TCP focused on whether simply lifting birth restrictions would motivate couples of childbearing age to give more births in China. Some claimed that TCP did not work well in China because the total fertility rate did not increase to a replacement level, while others observed a near-term increase in fertility intentions or birth rates following TCP implementation (Lau *et al*., 2018; Scharping, 2019; Meng and Lyu, 2022). Despite this, scholars remained concerned about the long-term sustainability of any demographic impact resulting from the relaxed birth restrictions (Zeng and Hesketh, 2016; Zhang *et al*., 2022).

Studies have explored the intention to have a second child in China after the TCP was implemented, with reported rates ranging from 13.2% to 69.3% (Bao *et al*., 2017; Wei *et al*., 2018; Li and Jiang, 2019; Zhu *et al*., 2022). However, these studies were mainly conducted in specific populations or settings, such as rural areas, women at outpatient gynaecology clinics or women from a single province. While a national survey indicated that 39.4% of Chinese women intended to have a second child following UTCP implementation, this intention to have a second child may not always lead to actual childbirth (Xu *et al*., 2016). The process from an intention to actual behaviour can be influenced by various factors, such as delayed fertility, decreased fecundity and work–family conflict. Most studies on birth rates and the number of births have been predictive, estimating an increase of 1 million to >10 million additional births per year (Zhai *et al*., 2017; Wenyao *et al*., 2019; Liu and Zhang, 2022). A previous study using national data suggested an immediate rise in births following the announcement of UTCP (Li *et al*., 2019). However, this analysis did not account for the selective TCP, and the study was limited to 2017, despite the TCP being in effect from 2011 to 2021.

With the three-child policy officially declared in August 2021, the controversy above still exists, as the essence of the three-child policy is still the relaxation of birth restrictions. A prevailing view is that if TCP was unable to encourage couples to give a second birth, the effectiveness of the three-child policy should also be questioned. Therefore, it is essential to understand both the short-term effects of the TCP and its long-term potential to inform future policymaking. Estimating additional birth rates or population increases by comparing the rates in China to those without the TCP may offer a comprehensive assessment of the TCP’s impact during its implementation. To fill the knowledge gap, our study collected population-level data from 1990 to 2020. We employed the quasi-experimental synthetic control method (SCM) and controlled interrupted time series analysis to retrospectively estimate the effect of the TCP on population growth.

## Methods

### Study design

The pre-TCP period (period before the enactment of the TCP) was set between 1990 and 2011. Besides, the post-TCP period was 2012–20, divided into three stages according to the year of policy shift, including 2012–13 for STCPI, 2014–15 for STCPII and 2016–20 for UTCP.

The design of our study is detailed in Supplementary Figure S1. To estimate the association between the TCP and demographic changes in China, the synthetic control method and a quasi-experimental comparative case study design were used. The synthetic control method is suitable for evaluating population-level interventions. It was proposed to predict what the birth rates and rates of natural population increase in China (the treated unit) would have been in the post-TCP period in the absence of TCP implementation (an estimate of the counterfactual outcome), through constructing a convex combination of control units weighted to match the pre-TCP ones in China as closely as possible (Abadie *et al*., 2010; 2015). More detailed explanations of the method, including its utility and implementation, have previously been published (Abadie, 2021).

For simplicity, we used ‘China’ to represent China’s mainland, as the TCP was only implemented in China’s mainland. Countries like Myanmar, Vietnam, Iran, Egypt and India, which had their own versions of a TCP between 2011 and 2020, were excluded from the donor pool countries (i.e. the pool of potential control units). Additionally, Hong Kong was retained in the donor poor due to its geographical proximity and shared socioeconomic characteristics with Mainland China. Consequently, our analysis included 123 countries or regions with the necessary data in the donor pool (further details are available in the Supplementary Methods).

### Data and measures

The outcomes were the rate of natural increase (also called natural increase in population) and the birth rate (referred to the crude birth rate). The birth rate was the number of births over a year divided by the total population at that year and was expressed as annual number of births per 1000 population. The rate of natural increase was calculated by the crude birth rate minus the crude death rate, which represented the proportion of population growth (or decline) determined exclusively by births and deaths and was expressed per 1000 population annually. The trend of the rate of natural population increase was expected to reflect the potential change of population structure, while the birth rate would be a direct reflection of the birth policy’s impact.

Covariates may play a relatively minor role in the analysis, but including covariates is able to result in a counterfactual that is structurally more similar to the treatment unit and reduce the risk of overfitting on random variability in large samples (Bonander, 2018). We included country-level factors associated with population growth in the model, including gross domestic product (GDP) per capita, life expectancy, mean years of schooling, the proportion of female aged 15–49 years and the proportion of urban population (details in the Supplementary Material).

Data for mean years of schooling were collected from the Human Development Data Center of United Nations Development Programme (Human Development Reports, 2022). Other country-level data were collected from the World Bank database (World Bank, 2022). Specifically, the data from the World Bank database were only a collection of China’s mainland not including the data of Hong Kong, Taiwan or Macao region. The final dataset covered the years from 1990 to 2020 and contained 123 comparison countries or regions. The list of comparison countries and regions included in the analysis is provided in Table S1 in the Supplementary Material.

### Statistical analysis

For each outcome, we created separate synthetic comparison groups, matching China with a weighted combination of untreated comparison countries based on pre-intervention rates and other country characteristics. Using the synthetic control method in a standard software routine (synth Stata module), we derived weights to countries in the donor pool to minimize the root mean squared prediction error (RMSPE) for the pre-TCP period with respect to the study outcomes and variables associated with them. The weighted mean of the control countries was used to estimate the birth rate (or the rate of natural increase) trend in the post-TCP period in the absence of TCP implementation. Details were in the Supplementary Methods.

To determine whether the difference in rates of natural population increase (or birth rates) between China and its synthetic control after 2011 was due to chance, we conducted ‘in-space’ placebo tests, iteratively assigning the intervention to each control country and repeating the synthetic control procedure. Countries with a poor pre-TCP fit, defined as having an MSPE greater than five times China’s MSPE, were then dropped. As we hypothesized that TCP would be associated with an increase in the rate of natural population increase (or the birth rate), the one-sided test would be appropriate. We calculated the test statistics as the mean post-TCP difference in the outcomes between China and synthetic China and derived the one-sided pseudo-*P*-value via dividing the number of countries with a difference equal to or more negative than China by the number of remaining placebo countries plus one. A total of 102 countries/regions were included in the pseudo-*P*-value calculation for the rate of natural population increase, and 106 countries/regions were included in the calculation for the birth rate.

Finally, we tested the associations of interest using controlled interrupted time series analysis, with synthetic China serving as the control group (Linden, 2015). This allowed for the quantification of the differences in both level and slope changes post-TCP implementation, which took the following form:


$$\begin{aligned} {Y_t} = & {\beta _0} + {\beta _1}T + {\beta _2}{X_t} + {\beta _3}T{X_t} + {\beta _4}G + {\beta _5}GT \\ &+ {\beta _6}G{X_t} + {\beta _7}G{X_t}T + {\varepsilon _t} \end{aligned}$$

${Y_t}$ is the rate of natural increase or the birth rate at time $t$; $T$ is a linear time trend; ${X_t}$ is a dummy variable for the intervention; and $G$ is a dummy variable for the treated (China) and control group (synthetic China). The coefficients of interest are ${\beta _6}$ and ${\beta _7}$. The former provides the estimated difference in the level change after TCP implementation between China and synthetic China, and the latter provides the estimated difference in the change in slope between the two groups after the policy went into effect. We used Newey–West CIs to account for autocorrelation.

### Sensitive analysis

The use of indicators immediately preceding the implementation of the TCP to create synthetic comparison groups might lead to overfitting concerns. To address this issue, we excluded data from 2010 when constructing an alternative synthetic China. We then repeated the analysis using this alternative synthetic China. It is noteworthy that countries like Myanmar, Vietnam, Iran, Egypt and India had or have implemented their TCPs, but their objective was to control birth numbers, in contrast to China’s policy aimed at increasing them. Excluding these countries from our analysis could potentially bias the estimation of the effect of China’s TCP, especially if they were assigned significant weights in the SCM model. To account for this, we included these countries in a separate analysis. By adding nations like Vietnam, which have differing birth control objectives, to the donor pool for our sensitivity analysis, we could more thoroughly test the robustness of our primary conclusions.

The analysis relied on the assumption that there were no spillover effects into one or more groups in the donor pool, especially if these received large weights. Therefore, we performed a leave-*k*-out analysis in which highly influential countries are iteratively removed from the donor pool. Specifically, this is performed iteratively so that each iteration reduces the donor pool by one (*k* = 1 in the first iteration, *k* = 2 in the second and so on) and refits the synthetic control model using the restricted donor pool, until the pre-intervention RMSPE is more than twice that of the main analysis. We then compared the similarity of demographic and economic characteristics between China and the countries that were assigned large weights. To further assess the sensitivity of our results to the composition of synthetic China, we performed a Monte Carlo simulation analysis. This involved randomly selecting the number of countries (ranging from 20 to 123) to include in the synthetic comparison, as well as the specific countries comprising the donor pool. We then repeated our analysis of the effect of TCPs on the outcomes using various comparison country sets. This simulation was performed over 500 iterations for each outcome of the study.

For additional robustness checks, we then applied single-group interrupted time series analysis as an alternative method (details in the Supplementary Methods). All statistical analyses were performed using Stata version 17.0 (StataCorp, College Station, Texas 77845, USA).

## Results

China had lower rate of natural population increase and lower birth rate throughout the study period compared with the mean rates in the donor pool (Supplementary Figure S2). The rate of natural increase dropped precipitously in China between 1990 and 2010, decreasing by 9.1 per 1000 population, while the mean rate in the donor pool had a smaller decline of 4.6 per 1000. Meanwhile, the birth rate dropped by 9.2 per 1000 in China and declined by 6.1 per 1000 in the donor pool. The three peaks of the birth rate after 2010 in China, as well as the rate of natural increase, were seen in 2012, 2014 and 2016. After then, the rate of natural increase and the birth rate in China declined sharply.

### Association between TCP and the rate of natural population increase

Seven countries in the donor pool were assigned non-zero weights and made up of synthetic China (Table S2 in the Supplementary Material). The covariate balance between China and synthetic China (Table 1) showed that variables were well matched, and the pre-TCP synthetic control model fit was reasonable, with the RMSPE = 0.196 (Table 2).

**Table 1.** Characteristics of China, all comparison countries and synthetic control groups during the pre-TCP period

		Synthetic China		
Characteristics	China	Rate of natural increase	Birth rate	Donor pool mean
Rate of natural increase (per 1000)	8.28 (0.65)	8.26 (3.21)		14.36 (0.96)
Birth rate (per 1000)	14.92 (0.64)		14.94 (2.36)	23.43 (1.03)
Ln (GDP per capita)	6.95 (1.78)	7.72 (0.43)	8.26 (0.43)	8.12 (0.14)
Life expectancy (years)	71.50 (0.38)	69.35 (3.71)	73.23 (0.86)	68.29 (0.89)
Urban population (% of total population)	36.82 (1.56)	41.50 (5.07)	49.97 (6.72)	57.10 (2.11)
Female aged 15–49 years (% of total population)	27.68 (0.85)	24.94 (0.28)	24.41 (0.75)	24.89 (0.16)
Mean years of schooling (years)	6.24 (0.16)	7.50 (0.85)	8.01 (0.80)	7.38 (0.27)
Number of countries or regions	1	7	6	123

**Table 2.** Estimates of the effects of TCP on the birth rate and the rate of natural increase

	Annual rate per 1000 residents	
Items	Rate of natural increase	Birth rate
China	5.07	12.16
Synthetic China	4.05	11.00
Rate difference	1.02	1.16
Percentage difference	25.0	10.5
Pseudo *P*-value*	0.020	0.047
Model fit (RMSPE)^a^	0.196	0.195

* A total of 102 countries/regions were included in the pseudo-*P*-value calculation for the rate of natural population increase, and 106 countries/regions were included in the calculation for the birth rate. Calculations for pseudo-*P*-value are presented in the Methods section.

a The pre-TCP RMSPE in China (the average of the squared discrepancies between China and its synthetic controls pre-TCP implementation).

**Figure 1.**
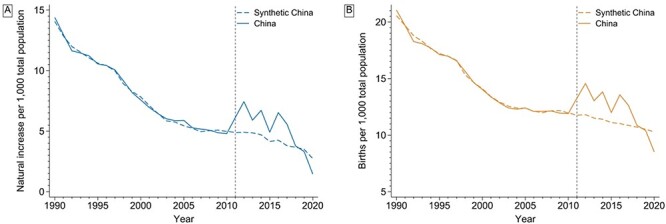
Rates of natural increase (a) and birth rates (b) in China and synthetic China

**Figure 2.**
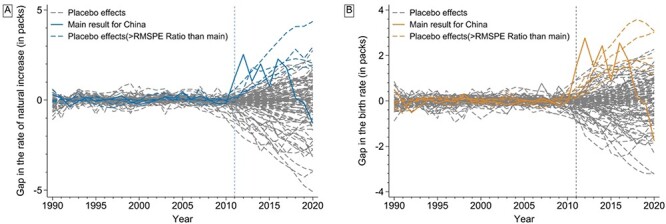
Placebo test results for the association between TCP implementation and the rate of natural increase (a) and the birth rate (b) in China.

The trend of the rate of natural increase in China appeared to be well matched before TCP implementation by synthetic China (Figure 1). After TCP implementation, the mean rate of natural increase in China (5.07 per 1000 population) was >25.0% than the synthetic control rate (4.05 per 1000 population) (Table 2). The placebo test results suggested that TCP was associated with a rise in the rate of natural increase in China (Table 2 and Figure 2), with a one-sided pseudo-*P*-value equal to 0.020. Gaps in rates of natural increase between China and its synthetic control were >2‰ in 2012, 2014 and 2016 but narrowed over time after 2016 (Table S3 in the Supplementary Material).

The statistical inference of the controlled interrupted time series analyses indicated a 2.262 per 1000 higher level in the rate of natural increase following TCP implementation in China than that in synthetic China [*P* = 0.021, 95% CI = (0.358, 4.165)] (Table 3). Meanwhile, the declining pre–post trend of natural increase rates in China was faster than that of its synthetic control group, with 0.274 more annual reduction per 1000 population [*P* = 0.028, 95% CI = (−0.518, −0.031)].

**Table 3.** The difference in level and slope change after TCP implementation, with the results from controlled interrupted time series analysis

	Rate of natural increase		Birth rate	
Items	Coef. (95% CI)	*P*-value	Coef. (95% CI)	*P*-value
Level change difference^a^	2.262 (0.358, 4.165)	0.021	2.788(0.644, 4.931)	0.012
Slope change difference^a^	−0.274 (−0.518, −0.031)	0.028	−0.340 (−0.584, −0.096)	0.007

a Difference is between China and synthetic China.

### Association between TCP and the birth rate

The birth rate synthetic control unit was composed of six countries assigned non-zero weights (Table S2 in the Supplementary Material). The covariate variables were well matched with the exception of the mean years of schooling (1.77 years difference) and the proportion of female aged 15–49 years (3.27% difference) (Table 1). However, the pre-TCP MSPE suggested a good model fit (Table 2). In the synthetic control analysis, the birth rate was >10.5% in China (12.16 per 1000) than synthetic China (11.00 per 1000) after TCP implementation (Table 2 and Figure 1). The placebo tests suggested that TCP was associated with a rise in the birth rate in China (Table 2 and Figure 2), with a one-sided pseudo-*P*-value = 0.047. Gaps in birth rates between China and its synthetic control were >2‰ in 2012, 2014 and 2016 but narrowed over time after 2016 (Table S3 in the Supplementary Material).

In the controlled interrupted time series analysis (Table 3), the birth rate in China was 2.788 per 1000 higher than its synthetic control [*P* = 0.012, 95% CI = (0.644, 4.931)] and the declining pre–post trend of birth rates in China was faster than that of its synthetic control group, with 0.340 more annual reduction per 1000 population [*P* = 0.007, 95% CI = (−0.584, −0.096)].

### Sensitivity analysis

In the sensitivity analysis using the alternative synthetic China constructed without 2010 data, model fit remained basically the same for the rate of natural increase and the birth rate (Tables S4 and S5 in the Supplementary Material). Differences in the outcomes between China and alternative synthetic China were distinguishable from chance variation, consistent with the results of main analysis. After incorporating the data from Myanmar, Vietnam, Iran, Egypt and India into the model, none of the five countries were assigned weights greater than zero in the synthetic control group. The results remained consistent with the main findings (Tables S6 and S7 in the Supplementary Material). Although several countries such as Albania and Croatia were assigned weights >70%, the results from leave-*k*-out analysis indicated that the synthetic control method produces robust outcomes for the two indicators, even when influential countries are excluded (Tables S8, S9 and Figure S3 in the Supplementary Material). In addition, the results from Monte Carlo simulation analysis suggested that as the model fit improved (i.e. the pre-TCP RMSPE decreased), the absolute values of the rate differences and level change difference tended to increase, while the change difference in slope decreased. The results implied that the specifications supporting a lower short-term effect or a sustained effect of the TCP tended to have poorer balance between the pre-TCP factors, undermining their validity (Supplementary Figures S4 and S5). The single-group interrupted time series analysis further corroborates our main findings, indicating a short-term effect of the TCP but not a long-term one (Supplementary Table S10).

## Discussion

To our knowledge, this is the first study to estimate birth rates and rates of natural increase for China in the absence of TCP implementation using the synthetic control method, which allows us to quantitatively evaluate the effect of lifting birth restrictions on population growth during the whole post-TCP period. By comparing post-TCP implementation rates in China with estimated rates in synthetic China, we found that TCP was associated with 2012–20’s population increase in China. However, rates of natural increase and birth rates declined faster in China than rates in the absence of TCP implementation from 2012 to 2020. These findings were robust to alternative model specifications.

As each policy relaxation was followed by a 1-year sharp increase in birth rates and rates of natural population increase, it meant three shifts of birth control policy all came into effect in the short term. Studies on the shift to the first stage of selective TCP were limited. To fill the gaps, this study estimated the birth rate in China was 2.76 per 1000 population higher than that in the absence of the policy in 2012. In addition, great importance was also ascribed to the impact of STCPII on the increase in births in this study, while a study from Shanghai found no evidence of it (Chen *et al*., 2022). However, the latter only collected data from one city, the results of which might not be generalized to other areas in China. Meanwhile, the results from a national study supported our findings, estimating the residents’ willingness of more children increased by 13.9% after STCPII (Meng and Lyu, 2022). As for the short-term effect of UTCP, a study revealed that UTCP was associated with an additional 5.40 (range 4.34–6.46) million births in the first 18 months of implementation, basically consistent with our estimation (by multiple the estimated rates with total population) (Li *et al*., 2019). The findings of an association between TCP implementation and population increase indicated a large amount of fertility desire repressed by the one-child policy was released, given the fact that the percentage of mothers >35 years old increased by 10–30% and the number of multiparous mothers had almost doubled after TCP implementation (Zhang *et al*., 2018; 2020; Liu *et al*., 2019).

However, both the birth rate and the rate of natural increase in China declined faster than its synthetic control during 2012–20, with an additional annual decrease per 1000 population of 0.340 and 0.274, respectively. Before the end of TCP, the rates in China were no longer higher than the estimated ones in the absence of the TCP scenario. The results suggested that the effect of TCP was diminishing fast in the late period of the policy and might not be sustained in the long run. As the one-child policy had already been relaxed for rural residents since the 1980s, the increase in fertility is mainly based on urban women’s response to TCP. However, women in urban areas have higher education levels, which is a well-known factor of low-fertility intentions (Chatterjee, 2020; Ghaznavi *et al*., 2022). Additionally, a previous study indicated that people who were born in the only-child family were less willing to have more children (Wang and Sun, 2020). It was also suggested that the women’s marriage age, the pecuniary costs of having children, family income, family support, having a firstborn son and so forth were significantly and negatively related to the desired fertility of Chinese women (Peng, 2020; Wang and Sun, 2020). Thus, the effects of socioeconomic factors on the rapid decline of the birth rate or the rate of natural increase might have far exceeded that of the relaxation of birth control policy. The unsustained impact of TCP on population growth might be a signal that China has dropped into the low-fertility trap, which refers to the phenomenon that it is difficult for a country to bring fertility up once it has already fallen to a low level (Lutz *et al*., 2006). It is worth noting that TCP was not enforced as strictly as the one-child policy. For example, fines have been nearly abolished even if some couples have three children or more, indicating that the essence of TCP was to encourage more births (Alpermann and Zhan, 2019). However, from our results, the effect of the policy with lifting birth restrictions alone seems impossible to boost China’s birth rates in the long run.

Financial challenges and the difficulty of balancing childrearing with work significantly influence fertility decisions among young Chinese parents (Jing *et al*., 2022; Kang *et al*., 2022). Insights from other countries’ experiences could inform Chinese birth policymakers. For example, to increase birth rates, Singapore implemented pronatalist policies like the baby bonus scheme, offering cash incentives to lessen the financial burden of raising children and promote larger families (Wong and Yeoh, 2003; Langridge *et al*., 2012; Wood and Neels, 2019). Nevertheless, the pronatalist polices are often perceived as providing short-term benefits with limited long-term effects (Hsiao‐Li Sun and Zhang, 2012). Hong Kong, Japan and the Republic of Korea have also provided financial support to families with children and expanded childcare services (Sobotka *et al*., 2019). However, financial incentives have had minimal impact on Hong Kong’s birth rates (Research Office, 2022). In the Republic of Korea, the fertility rate dropped to a global low of 0.81 in 2021. In contrast, Japan appears to have a stable fertility rate over the past decade. Assessing the direct impact of government support measures on birth rates is challenging, but studies suggest that sustained efforts to improve access to education and healthcare have long-term effects on birth rates (Straughan, 2008; Teo, 2010). For instance, Singapore has further implemented a parent-government co-saving scheme to cover children’s educational and medical expenses and introduced subsidized housing sales policies. In Japan, policies like parental leave and child benefits have been well received as evidenced by increased take-up rates and benefit payments (Bradshaw and Tokoro, 2014). In contrast to East Asian countries, Nordic countries and most Western European countries allocate 3–4% of their GDP to family services, particularly childcare, to support higher fertility rates (Sobotka *et al*., 2019). The experience from these developed countries shows that policies supporting children and family welfare are crucial. However, the effectiveness of childbearing-encouraging policies can be undermined by a mismatch between people’s expectations and the available initiatives (Hsiao‐Li Sun and Zhang, 2012). In extreme cases, policies that aim to compel childbearing behaviour rather than to enhance intrinsic motivation may eventually lower people’s sense of autonomy and lead to a reliance on external factors for fertility decisions (Botev, 2015). As pronatalist policies are often embedded in a wider institutional and cultural context, further research is needed to determine the applicability of policies and strategies implemented in other countries to the specific context of China.

The credibility of this study relies on the comparability between China and the synthetic control group. From our SCM model, Albania and Croatia were assigned large weights in the synthetic control group due to their similarity to China after combining their demographic and socioeconomic statistics during 1990–2010. This similarity might stem from the extensive societal and economic restructuring in Albania and Croatia at the end of the 20th century (Lerch, 2013; Jemna and David, 2022), as well as various economic reforms in China (Lu *et al*., 2019). Previous studies demonstrated that regardless of the specific form, economic development caused by political changes or social reforms was linked to a decline in the fertility rates (Fanati and Manfredi, 2003; Sobotka *et al*., 2011; Wang and Sun, 2016). It is essential to note that neither Albania’s higher average population growth rate nor Croatia’s lower one, compared to China, makes them standalone comparators for China (World Bank, 2022). Additionally, in some aspects of socioeconomic characteristics, like GDP per capita, China ranks between Croatia and Albania (Human Development Reports, 2022; World Bank, 2022). This suggests that using a weighted combination of several countries, including Albania and Croatia, may mimic a similar context to China. Moreover, since the late 20th century, global economic and cultural exchanges have intensified (Kalemli-Ozcan *et al*., 2013). Some scholars claimed that the demographic shifts could result from the diffusion of Western norms and attitudes towards family and childbearing in developing countries (Lerch, 2014; Hendi, 2017). Increasing domestic and international migration may be leading to a convergence in fertility perspectives among young people in middle- and high-income countries, like China, Albania and Croatia (Lerch, 2014; Ileš and Karačić, 2022; Dong *et al*., 2023). However, further research is needed to explore the impact of socioeconomic reforms and migration on fertility transitions in various socioeconomic contexts.

As we noted earlier, constructing a realistic ‘counterfactual’ for China was challenging due to the uniqueness of its one-child policy implementation. Debates on the role of the one-child policy in China’s demographic changes have persisted with inconsistent results (Zhang, 2017; Scharping, 2019). A recent study suggested that the trajectory of China’s fertility transition and total population growth would have been statistically very similar to the observed pattern over the past three decades in the context of no one-child policy (Gietel-Basten *et al*., 2019). Many scholars believed that rapid economic development alone would have reduced fertility substantially, as has been the case in many other developing countries (Lerch, 2013; Zeng and Hesketh, 2016; Aitken, 2022). Therefore, an implicit assumption in our use of the SCM approach is that, during the pre-TCP period, social and economic changes were the primary drivers of fertility decline rather than the one-child policy itself. It is crucial to note that the primary objective of our study was not to find a replica of China’s policy background. Instead, the constructed synthetic control group was based on their overall demographic and socioeconomic similarities to China.

Our study has limitations. While we adjusted for some socioeconomic variables in our models, eliminating confounding factors is unlikely. For instance, the discrepancy between people’s expectations and the policies available could also contribute to the decline in fertility (Sobotka *et al*., 2019). Future research with access to extensive individual-level data might provide further insights into this issue. Additionally, the TCP was not the sole factor influencing changes in birth rates or natural population growth during the study period. Other shifts in family planning policies, population risk factors and medical care might have also played a role, both in China and in the comparison countries. Our study, while providing insights into China’s demographic changes, faces limitations regarding its external validity and generalizability. The unique sociopolitical context of China, particularly in terms of its birth control policies, means that our findings may not be directly applicable to countries with different sociopolitical environments. Specifically, the impact of lifting birth restrictions in China may not mirror those in other contexts, where easing birth restrictions could align more closely with public expectations and help sustain fertility rates. Furthermore, our findings could be confounded by various programmes in the comparison countries. However, when compared to the general changes that perhaps affected by other reforms or strategies noted in comparable countries, the TCP did not seem to generate persistent and incremental improvements in population growth. Our preferred synthetic control approach effectively created comparison groups closely resembling China. This approach considered other factors while evaluating the impact of the TCP. In addition, we performed a series of sensitivity analyses, such as removing influential countries from the set of controls and randomly selecting different components of the donor pool. These additional analyses yielded consistent and robust results.

## Conclusions

Our research raises questions about whether the relaxation of birth restrictions is a viable method to promote population growth. We believed that the step of lifting birth restrictions was a necessary and highly desirable action, allowing large amounts of couples to have the number of children they want. In our analysis, we confirmed the short-term effect of TCP on population increase, but lifting birth restrictions alone was not effective in the long run. For countries that dropped into the low-fertility trap, many efforts need to be made. Japan and Korea had implemented a series of family planning policies to curb the rapid growth of population during 1950–80s but shifted to birth incentive policies when governments observed the trend of low fertility (Zeman *et al*., 2018). However, low-fertility intention remains an urgent problem in these countries. Hence, we support the call for cancelling any birth restrictions in China and the government should make attempts to construct various financial supportive systems as quickly as possible, to encourage more births of a family, before sinking deeper into the low-fertility trap. While many countries have explored financial support strategies to encourage higher birth rates, the effectiveness of such policies significantly varies depending on the country’s specific socioeconomic context. Further research focusing on the applicability and impact of birth incentive policies within China’s specific context may offer valuable insights for the formulation and optimization of future birth policies in China.

## Supplementary Material

czae008_Supp

## Data Availability

All data sources used in this study are publicly available online. Data from the World Bank database and Human Development Reports can be found at https://data.worldbank.org/ and https://hdr.undp.org/data-center, respectively.

## Abbreviations

TCP = two-child policy

STCPI = the first phase of the selective two-child policy

STCPII = the second phase of the selective two-child policy

UTCP = universal two-child policy

RMSPE = root mean squared prediction error

## Reflexivity statement

This article presents research conducted by a diverse group of researchers in terms of gender identity and seniority. This article includes four authors who identify as women and two who identify as men. Two of the authors specialize in health policy evaluation and health economics research, and two authors’ research interests centre around the changes of birth policy and family planning policy and their impacts on social medicine problems, ranging from demographic changes to reproductive health. Several team members also have experience working for Sichuan Health and Family Planning Commission, which brings additional perspectives to the research. The research team has multidisciplinary expertise in health economics, policy analysis, public health and social science. All authors have extensive experience with quantitative research and health policy research in China.
