# Supplementary material for: The effect of gradually lifting the two-child policy on demographic changes in China
Source: Health Policy Plan. 2024 Feb 9;39(4):363–71. doi: 10.1093/heapol/czae008 (PMC11005836; doi:10.1093/heapol/czae008)
Supplement: czae008_Supp [file czae008_supp.zip › suppl_data/Supplementary Materials_30Jan2024.pdf]

Supplementary Materials

Article title: The effect of gradually lifting the two-child policy on demographic changes in China

Contents

Supplementary Methods 3

Details in synthetic control method 3

Selecting Comparison Countries and Regions for the Donor Pool........ 4

County-Level Predictor Details........ 4

Single-group interrupted time series analysis 5

References........ 8

Supplementary Tables 9

Table S1 The List of Comparison Countries and Regions 9

Table S2 Donor Pool Weights by Model 10

Table S3 Rates of population increase and birth rates post-TCP based on the results from synthetic control method 11

Table S4 Synthetic Control Results, Sensitivity Analysis: excluding the data of 2010 in the model. 12

Table S5 Controlled Interrupted time series results, Sensitivity Analysis: excluding the data of 2010 in the model. 13

Table S6 Synthetic Control Results, Sensitivity Analysis: including the data of Myanmar, Vietnam, Iran, Egypt and India. 14

Table S7 Controlled Interrupted time series results, Sensitivity Analysis: including the data of Myanmar, Vietnam, Iran, Egypt and India. 15

Table S8 Results for the rate of natural increase, from the leave-*k*-out analysis that iteratively reduced the donor pool by excluding the most influential country or region from the synthetic control unit (Continued). 16

Table S8 Results for the rate of natural increase, from the leave-*k*-out analysis that iteratively reduced the donor pool by excluding the most influential country or region from the synthetic control unit. 17

Table S9 Results for the birth rate, from the leave-*k*-out analysis that iteratively reduced the donor pool by excluding the most influential country or region from the synthetic control unit. 19
Table S10 Single-group interrupted time series analyses for demographic changes in China. 20
Supplementary Figures 21
Figure S1 Flow chart of the study design. 21
Figure S2 Annual rate of natural population increase and annual birth rate in donor pool countries and in China (Note: Three vertical dotted lines denote the start year of each relaxation of birth restrictions respectively.) 22
Figure S3 Results from the leave-*k*-out analysis that iteratively reduced the donor pool by excluding the most influential country or region from the synthetic control unit. 23
Figure S4 Scatterplot and fitted quadratic relationship between RMSPE and the estimated value of rate difference(A), level change(B) and slope change difference(C) from simulation analysis of the relationship between the composition of the synthetic control and the effect of TCP on the rate of natural increase. 24
Figure S5 Scatterplot and fitted quadratic relationship between RMSPE and the estimated value of rate difference(A), level change(B) and slope change difference(C) from simulation analysis of the relationship between the composition of the synthetic control and the effect of TCP on the birth rate. .. 25

Supplementary Methods

Details in synthetic control method

We used the synthetic control method (SCM) to compare post-intervention outcomes between China and their estimated counterfactual comparison groups. SCM is suitable for estimating the effects of interventions in a limited number of treated units (Abadie *et al.*, 2010). Unlike common policy evaluation methods like the difference-in-differences approach, SCM generates a synthetic pre-intervention and post-intervention counterfactual. This method circumvents the often-unrealistic parallel trends assumption required by the difference-in-differences method (Abadie *et al.*, 2015). The synthetic control, or estimated counterfactual, is a weighted average of outcomes in untreated comparison or donor units (regions without a similar policy). Donor units are selected to maximize the preintervention fit of the synthetic control to the observed outcomes in the treated unit. To assess fit, we evaluated the root mean square prediction error (RMSPE) of the synthetic control. The lower the RMSPE, the better the fit in the preintervention period (Abadie *et al.*, 2010). The weighted mean of the donor units is then used to estimate the outcome trend in the postintervention period in the absence of the policy change (the counterfactual). Given our hypothesis that the two-child policy (TCP) would lead to increased birth rates and natural increase rates, a one-sided test would be appropriate. The analyses were performed using Stata, version 17.0 (StataCorp LLC), utilizing the synth and synth_runner commands with nested option.

For each outcome, we created separate synthetic comparison groups by matching China with a weighted combination of the comparison countries without similar intervention. This matching was based on pre-intervention rates and other country characteristics. Once we obtained well-fitting synthetic controls for China, we compared the post-TCP rates of China with its synthetic control. The specific weightings for each outcome's synthetic control are detailed in Table S2. The foundational assumption for the causal validity of our synthetic control design is that if China had not implemented the two-child policy in 2011, its average demographic trends would have closely aligned with those of the synthetic control group. Consequently, any divergence in demographic changes between China and the synthetic control group post-implementation can be

attributed to the relaxation of birth restrictions. The reliability of this assumption hinges on two key factors: firstly, the presence of sufficient untreated units to construct a synthetic control group that reliably follows the historical trends of the treated unit prior to the intervention; and secondly, the absence of unobserved confounders that might cause future demographic divergences between the treated unit and the synthetic control comparisons (Abadie, 2021).

Selecting Comparison Countries and Regions for the Donor Pool

Data from Human Development Data Center of United Nations Development Programme included 190 countries or regions (Human Development Reports, 2022), while data from World Bank included 219 countries or regions (World Bank, 2022). After matching the two databases with each other, 189 countries or regions were included in the dataset. We then excluded Myanmar, Vietnam, Iran, Egypt and India from the donor pool, because these countries have or had “two-child policy” during 2012-2020. The data for China in this study was only a collection of the data from China’s mainland. China has implemented the “One Country, Two Systems” policy in both Hong Kong and Macao, and Taiwan also has a similar system with Hong Kong. Therefore, Hong Kong may have a good representation for Macao and Taiwan. Although, we had no access to the data of mean years of schooling in Macao and Taiwan, we retained Hong Kong in the donor pool. Thus, there were 123 comparison countries or regions with the data needed in the final donor pool for analysis.

Although we cannot compare the effect of the two-child policy (TCP) with no intervention in the comparison groups, we can compare it with the general improvements—perhaps encouraged by other family planning policies or reforms—that were noted in comparable countries. By analogy with terminology from clinical trials, we do not interpret our results as a comparison between treatment and no treatment study arms, but rather between treatment and usual care.

County-Level Predictor Details

We included country-level variables associated with population growth in the model, including gross domestic product (GDP) per capita, life expectancy, mean years of

schooling, proportion of female ages 15-64 and proportion of urban population. Only mean years of schooling was collected from Human Development Data Center of United Nations Development Programme, which was calculated by the average number of completed years of education of a country's population aged 25 years and older, excluding years spent repeating individual grades. This predictor indicates great shares of the adult population according to the highest level of education attained or completed, and reflects a performing educational system. GDP per capita, life expectancy at birth, proportion of female ages 15-49 and proportion of urban population were collected from World Bank (Worldwide Governance Indicators).

GDP per capita was transformed with logarithmic in the synthetic control method, showing country's GDP divided by its total population (GDP is the sum of gross value added by all resident). Life expectancy at birth used here is the average number of years a newborn is expected to live if mortality patterns at the time of its birth remain constant in the future. It reflects the overall mortality level of a population and summarizes the mortality pattern that prevails across all age groups in a given year. Urban population refers to people living in urban areas as defined by national statistical offices. Proportion of urban population represented the number of persons residing in an area defined as "urban" per 100 total population, which was calculated by the Statistics Division of the United Nations Department of Economic and Social Affairs. Female population between the ages 15 to 49 as a percentage of the total population, which was related to the number of women at childbearing age. All the interpretation of these predictor were from the relative data sources.

Single-group interrupted time series analysis

In the main analyses, we conducted controlled interrupted time series analyses with synthetic China serving as the control group. As an alternative method, we then conducted a single-group interrupted time series analysis for each study outcome. The results were shown in Supplementary Table S10. Single-group interrupted time-series analysis is a popular evaluation strategy for observational data in which a single unit is studied, the dependent variable is a serially ordered time series, and multiple

observations are captured in both the pre- and post-intervention periods (Linden, 2015). The study design is called an interrupted time series because the intervention is expected to *interrupt* the level and/or trend of the time series, subsequent to its introduction.

In our study, single-group interrupted time-series analysis was implemented using linear regression of the birth rates or the rates of natural increase as the dependent variable and, as independent variables, (i) year of the study period from 1990 to 2011 as a continuous variable, (ii) a dichotomous variable to discern the pre- and post-intervention period (1990-2011 and 2012–2020, respectively), and (iii) year since onset of the TCP period with a value of zero for the pre-intervention period. The analysis took the following form:

$$Y_t = \beta_0 + \beta_1 T + \beta_2 X_t + \beta_3 T X_t + \varepsilon_t$$

Y_t is the rate of natural increase or the birth rate at time t; T is a linear time trend; X_t is a dummy variable for the intervention; β_1 represents the slope in the pre-intervention period; β_2 represents the level effect, the immediate change following the onset of the two-child policy; β_3 represents the change in slopes between the pre- and the post-TCP period. Regression diagnostics did not reveal major deviation of residuals from normal distribution. Fitted and predicted mean rates with their confidence intervals as well as prediction intervals were derived from the single-group interrupted time-series model. Single-group interrupted time-series analysis relies on the assumption that without intervention (counterfactual), the level and trend in outcome would remain unchanged throughout the study period provided that other factors affecting the outcome remain unchanged or change slowly (Linden, 2015). Despite that single-group interrupted time-series analysis may be prone to internal validity threats, this approach is still widely used to assess the impact of public health interventions when a comparable control group is not available (Barrio *et al.*, 2019).

The results showed before 2011, rate of natural increase seemed to decline significantly by -0.459 per 1,000 population (95%CI: -0.542 to -0.396; $p < 0.001$; Supplementary Table S10). In the first year of the two-child policy, rate in population

increase appeared to have increased significantly by 4.325 per 1,000 (95%CI: 2.764 to 5.878; $p < 0.001$). Whereas there were no differences in the trend of rate in population increase between the post-TCP period and the pre-TCP period (mean slope difference: -0.036; 95%CI: -0.269 to 0.197; $p = 0.754$).

Furthermore, the results showed before 2011, China's birth rate seemed to decline significantly by -0.451 per 1,000 population (95%CI: -0.544 to -0.358; $p < 0.001$; Supplementary Table S10). In the first year of the two-child policy, rate in population increase appeared to have increased significantly by 4.624 per 1,000 (95%CI: 2.834 to 6.413; $p < 0.001$). Whereas there were no differences in the trend of rate in population increase between the post-TCP period and the pre-TCP period (mean slope difference: -0.062; 95%CI: -0.296 to 0.172; $p = 0.591$).

References

Abadie A. 2021. Using synthetic controls: Feasibility, data requirements, and methodological aspects. *Journal of Economic Literature* **59**: 391–425.

Abadie A, Diamond A, Hainmueller J. 2010. Synthetic control methods for comparative case studies: Estimating the effect of california's tobacco control program. *Journal of the American Statistical Association* **105**: 493–505.

Abadie A, Diamond A, Hainmueller J. 2015. Comparative politics and the synthetic control method: COMPARATIVE POLITICS AND THE SYNTHETIC CONTROL METHOD. *American Journal of Political Science* **59**: 495–510.

Barrio G, Belza MJ, Carmona R, Hoyos J, Ronda E, Regidor E. 2019. The limits of single-group interrupted time series analysis in assessing the impact of smoke-free laws on short-term mortality. *International Journal of Drug Policy* **73**: 112–20.

Human Development Reports. 2022. *Human Development Reports*.

Linden A. 2015. Conducting interrupted time-series analysis for single- and multiple-group comparisons. *The Stata Journal: Promoting communications on statistics and Stata* **15**: 480–500.

World Bank. 2022. *World Bank Open Data*.

Supplementary Tables

Table S1 The List of Comparison Countries and Regions

ID	Name	ID	Name	ID	Name	ID	Name
1	Albania	32	Algeria	63	Kuwait	94	Poland
2	United Arab Emirates	33	Ecuador	64	Liberia	95	Portugal
3	Argentina	34	Spain	65	Libya	96	Paraguay
4	Armenia	35	Estonia	66	Sri Lanka	97	Qatar
5	Australia	36	Finland	67	Lesotho	98	Romania
6	Austria	37	Fiji	68	Lithuania	99	Russian Federation
7	Azerbaijan	38	France	69	Luxembourg	100	Rwanda
8	Burundi	39	Gabon	70	Latvia	101	Saudi Arabia
9	Belgium	40	United Kingdom	71	Morocco	102	Sudan
10	Benin	41	Ghana	72	Maldives	103	Senegal
11	Bangladesh	42	Guinea	73	Mexico	104	Singapore
12	Bulgaria	43	Greece	74	Mali	105	Sierra Leone
13	Bahrain	44	Guatemala	75	Malta	106	El Salvador
14	Belarus	45	Guyana	76	Mongolia	107	Slovenia
15	Belize	46	Hong Kong	77	Mozambique	108	Sweden
16	Brazil	47	Honduras	78	Mauritania	109	Syrian Arab Republic
17	Barbados	48	Croatia	79	Mauritius	110	Togo
18	Brunei Darussalam	49	Haiti	80	Malawi	111	Thailand
19	Botswana	50	Hungary	81	Malaysia	112	Tajikistan
20	Central African Republic	51	Indonesia	82	Namibia	113	Tonga
21	Canada	52	Ireland	83	Niger	114	Trinidad and Tobago
22	Switzerland	53	Iraq	84	Nicaragua	115	Tunisia
23	Chile	54	Iceland	85	Netherlands	116	Uganda
24	Cameroon	55	Israel	86	Norway	117	Ukraine
25	Colombia	56	Italy	87	Nepal	118	Uruguay
26	Costa Rica	57	Jamaica	88	New Zealand	119	United States
27	Cuba	58	Jordan	89	Pakistan	120	Samoa
28	Cyprus	59	Japan	90	Panama	121	South Africa
29	Germany	60	Kazakhstan	91	Peru	122	Zambia
30	Denmark	61	Kenya	92	Philippines	123	Zimbabwe
31	Dominican Republic	62	Cambodia	93	Papua New Guinea		

Table S2 Donor Pool Weights by Model

Country	Weight	Country	Weight
**Rate of natural increase**			
Albania	0.370	Rwanda	0.037
Croatia	0.353	Romania	0.017
Lesotho	0.121	Honduras	0.001
Maldives	0.100		
**Birth rate**			
Albania	0.304	Maldives	0.134
Croatia	0.405	Qatar	0.068
Kuwait	0.002	Romania	0.085

* The weights of the countries or regions that were not listed in the table were assigned zero.

Table S3 Rates of population increase and birth rates post-TCP based on the results from synthetic control method

Year		Rate of natural increase (per 1,000)			Birth rate (per 1,000)		
		China	Synthetic China	Rate Difference	China	Synthetic China	Rate Difference
STCP Ⅰ	2012	7.44	4.90	2.54	14.57	11.78	2.79
	2013	5.90	4.85	1.05	13.03	11.48	1.55
STCP Ⅱ	2014	6.71	4.70	2.01	13.83	11.38	2.45
	2015	4.92	4.14	0.78	11.99	11.09	0.9
UTCP	2016	6.53	4.25	2.28	13.57	11.00	2.57
	2017	5.58	3.79	1.79	12.64	10.83	1.81
	2018	3.78	3.66	0.12	10.86	10.69	0.17
	2019	3.32	3.49	-0.17	10.41	10.50	-0.09
	2020	1.45	2.72	-1.27	8.52	10.27	-1.75

TCP = two-child policy; STCP = selective two-child policy; UTCP = universal two-child policy

Table S4 Synthetic Control Results, Sensitivity Analysis: excluding the data of 2010 in the model.

	Annual rate per 1000 residents	
	Rate of natural increase	Birth rate
China	5.070	12.16
Synthetic China	3.887	10.98
Rate difference	1.183	1.18
% difference	30.4	10.7
Pseudo *P* value*	0.029	0.047
Model fit (RMSPE) #	0.199	0.196

RMSPE = root mean squared prediction error;

*102 countries and regions were included in the pseudo-*P* value calculation for the rate of natural increase and 106 countries/regions were included in the calculation for the birth rate. Calculations for pseudo-P value are presented in the Methods section. #The pre-intervention RMSPE in China (the average of the squared discrepancies between China and its synthetic controls before 2011).

Table S5 Controlled Interrupted time series results, Sensitivity Analysis: excluding the data of 2010 in the model.

	Rate of natural increase		Birth rate	
	***β*(95%CI)**	***P***	***β*(95%CI)**	***P***
Level change difference*	2.307 (0.423, 4.191)	0.017	2.791(0.671, 4.911)	0.011
Slope change difference*	-0.257(-499, -0.150)	0.038	-0.352(-0.595, -0.109)	0.005

CI = confidence interval;

*Difference is between China and synthetic China.

Table S6 Synthetic Control Results, Sensitivity Analysis: including the data of Myanmar, Vietnam, Iran, Egypt and India.

	Annual rate per 1000 residents	
	**Rate of natural increase**	**Birth rate**
China	5.07	12.16
Synthetic China	4.06	11.02
Rate difference	1.01	1.14
% difference	24.9	10.3
Pseudo *P* value*	0.037	0.045
Model fit (RMSPE) #	0.196	0.194

RMSPE = root mean squared prediction error;

*107 countries/regions were included in the pseudo-*P* value calculation for the rate of natural population increase and 110 countries/regions were included in the calculation for the birth rate. Calculations for pseudo-P value are presented in the Methods section. #The pre-TCP RMSPE in China (the average of the squared discrepancies between China and its synthetic controls pre-TCP implementation).

Table S7 Controlled Interrupted time series results, Sensitivity Analysis: including the data of Myanmar, Vietnam, Iran, Egypt and India.

	Rate of natural increase		Birth rate	
	***β*(95%CI)**	***P***	***β*(95%CI)**	***P***
Level change difference*	2.260 (0.356, 4.163)	0.021	2.786 (0.641, 4.931)	0.012
Slope change difference*	-0.274 (-0.518, -0.031)	0.028	-0.340 (-0.584, -0.096)	0.007

CI = confidence interval;

*Difference is between China and synthetic China.

Table S8 Results for the rate of natural increase, from the leave-*k*-out analysis that iteratively reduced the donor pool by excluding the most influential country or region from the synthetic control unit (Continued).

	Main	Iteration 1	Iteration 2	Iteration 3	Iteration 4	Iteration 5
Excluded countries	None	Albania	+Lesotho	+Costa Rica	+Thailand	+Croatia
Rate difference	1.02	0.51	1.48	1.3	1.1	0.65
% difference	25.0	11.2	41.3	34.5	27.6	14.6
RMSPE	0.196	0.208	0.269	0.286	0.295	0.373
No. of donor pool	123	122	121	120	119	118
Synthetic control, Name(Weight)	Albania (0.370)	Lesotho (0.401)	Costa Rica (0.552)	Thailand (0.253)	Croatia (0.301)	Malaysia (0.399)
	Croatia (0.353)	Croatia (0.364)	Croatia (0.306)	Croatia (0.242)	Malaysia (0.282)	Ukraine (0.235)
	Lesotho (0.121)	Romania (0.086)	Lithuania (0.126)	Malaysia (0.225)	United Arab Emirates (0.173)	Singapore (0.128)
	Maldives (0.100)	United Arab Emirates (0.052)	Ukraine (0.016)	Lithuania (0.127)	Lithuania (0.169)	Japan (0.093)
	Rwanda (0.037)	Qatar (0.041)	-	United Arab Emirates (0.083)	Maldives (0.064)	Lithuania (0.059)
	Romania (0.017)	Maldives (0.039)	-	Maldives (0.070)	Ukraine (0.011)	United Arab Emirates (0.053)
	Honduras (0.001)	Rwanda (0.018)	-	-	-	Qatar (0.034)

Table S8 Results for the rate of natural increase, from the leave-*k*-out analysis that iteratively reduced the donor pool by excluding the most influential country or region from the synthetic control unit.

	Iteration 6	Iteration 7	Iteration 8	Iteration 9	Iteration 10
Excluded countries	+Malaysia	+El Salvador	+South Africa	+Malta	+Japan
Rate difference	0.8	0.58	1.23	1.53	0.96
% difference	18.6	12.9	32.1	43.3	23.5
RMSPE	0.392	0.407	0.413	0.416	0.422
No. of donor pool	117	116	115	114	113
Synthetic control, Name(Weight)	El Salvador(0.394)	South Africa(0.287)	Malta(0.241)	Japan(0.250)	Singapore(0.338)
	Ukraine(0.241)	Japan(0.219)	Singapore(0.176)	Singapore(0.222)	Ukraine(0.181)
	Singapore(0.197)	Qatar(0.169)	Maldives(0.163)	Maldives(0.160)	Nepal(0.162)
	Lithuania(0.093)	Singapore(0.156)	Qatar(0.144)	Lithuania(0.153)	Lithuania(0.122)
	United Arab Emirates(0.045)	Lithuania(0.115)	Japan(0.123)	Nepal(0.141)	Maldives(0.073)
	Japan(0.022)	Malta(0.050)	Lithuania(0.089)	Ukraine(0.062)	Qatar(0.067)
	Qatar(0.009)	Maldives(0.004)	Ukraine(0.046)	Namibia(0.007)	Cyprus(0.058)
	-	-	Nepal(0.019)	Qatar(0.005)	-

RMSPE = root mean squared prediction error;

Table S9 Results for the birth rate, from the leave-*k*-out analysis that iteratively reduced the donor pool by excluding the most influential country or region from the synthetic control unit (Continued).

	Main	Iteration 1	Iteration 2	Iteration 3	Iteration 4
Excluded countries	None	Croatia	+Germany	+Albania	+Singapore
Rate difference	1.16	0.46	0.59	0.93	0.94
% difference	10.5	3.9	5.1	8.3	8.4
RMSPE	0.194	0.264	0.293	0.319	0.357
No. of donor pool	123	122	121	120	119
Synthetic control, Name(Weight)	Croatia (0.405)	Germany (0.517)	Albania (0.459)	Singapore (0.253)	Malta (0.393)
	Albania (0.304)	Malaysia (0.152)	Romania (0.423)	Malta (0.250)	United Arab Emirates (0.171)
	Maldives (0.134)	Maldives (0.150)	Malaysia (0.070)	Romania (0.202)	Romania (0.167)
	Romania (0.085)	Albania (0.144)	Maldives (0.028)	Malaysia (0.148)	Malaysia (0.137)
	Qatar (0.068)	Singapore (0.037)	Malta (0.014)	Maldives (0.088)	Cyprus (0.070)
	Kuwait (0.002)	-	Singapore (0.005)	United Arab Emirates (0.059)	Maldives (0.062)
	-	-	Slovenia (0.001)	-	-

RMSPE = root mean squared prediction error;

Table S9 Results for the birth rate, from the leave-*k*-out analysis that iteratively reduced the donor pool by excluding the most influential country or region from the synthetic control unit.

	Iteration 5	Iteration 6	Iteration 7	Iteration 8	Iteration 9
Excluded countries	+Malta	+Cyprus	+United Arab Emirates	+Japan	+Thailand
Rate difference	1.06	1.31	1.60	1.17	1.11
% difference	9.5	12.1	15.1	10.6	10.0
RMSPE	0.389	0.418	0.448	0.450	0.457
No. of donor pool	118	117	116	115	114
Synthetic control, Name(Weight)	Cyprus (0.572)	United Arab Emirates (0.354)	Japan (0.448)	Thailand (0.475)	Cuba (0.226)
	United Arab Emirates (0.205)	Japan (0.231)	Maldives (0.224)	Lithuania (0.228)	Qatar (0.186)
	Romania (0.149)	Malaysia (0.143)	Qatar (0.144)	Cuba (0.138)	Lithuania (0.183)
	Malaysia (0.074)	Poland (0.092)	Lithuania (0.137)	Maldives (0.100)	Switzerland (0.173)
	-	Lithuania (0.077)	Malaysia (0.047)	Qatar (0.059)	Maldives (0.156)
	-	Romania (0.039)	-	-	Poland (0.075)
	-	Maldives (0.036)	-	-	-
	-	Hongkong (0.028)	-	-	-

RMSPE = root mean squared prediction error;

Table S10 Single-group interrupted time series analyses for demographic changes in China.

	Rate of natural increase		Birth rate	
	***β*(95%CI)**	***P***	***β*(95%CI)**	***P***
Time	-0.469 (-0.542, -0.396)	<0.001	-0.451 (-0.544, -0.358)	<0.001
Level effect*	4.325 (2.764, 5.887)	<0.001	4.624 (2.834, 6.413)	<0.001
Slope change #	-0.036 (-0.269, 0.197)	0.754	-0.062 (-0.296, 0.172)	0.591

CI = confidence interval;

* The immediate change following the onset of the two-child policy (TCP);

# The change in slopes between the pre- and the post-TCP period.

Supplementary Figures

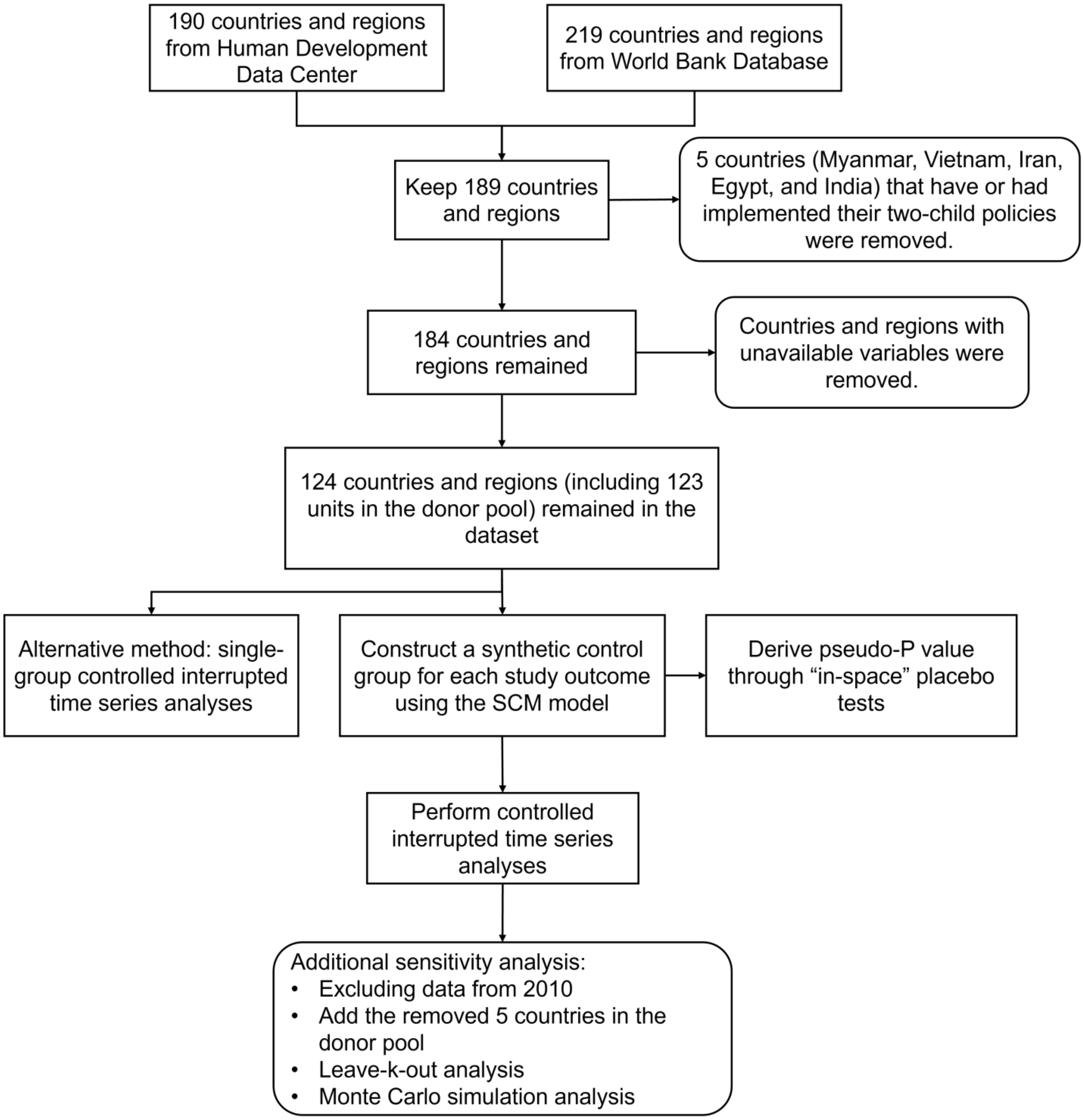

Figure S1 Flow chart of the study design.

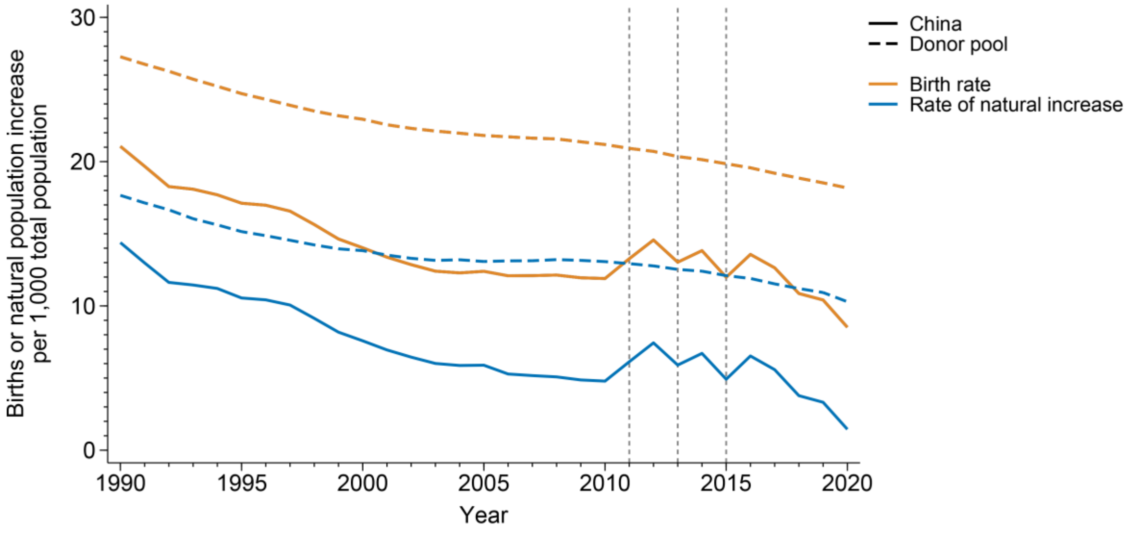

Figure S2 Annual rate of natural population increase and annual birth rate in donor pool countries and in China (Note: Three vertical dotted lines denote the start year of each relaxation of birth restrictions respectively.)

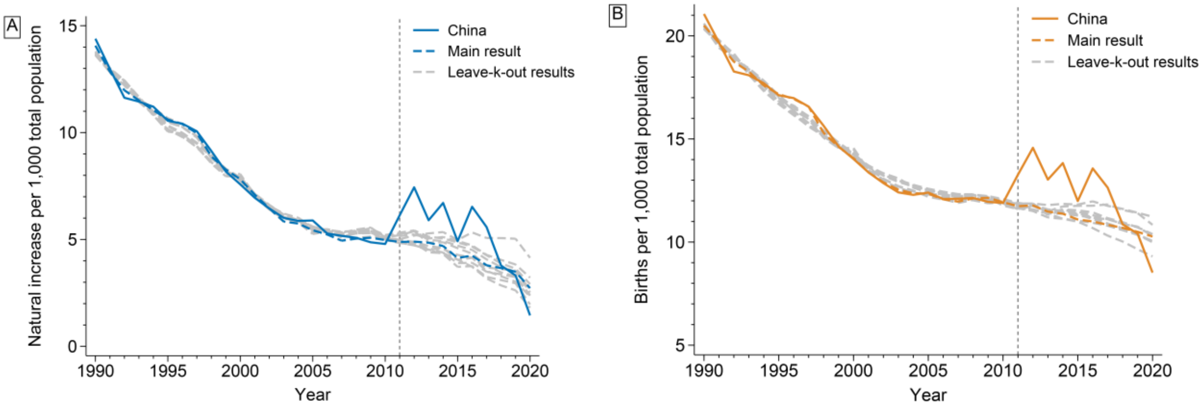

Figure S3 Results from the leave-*k*-out analysis that iteratively reduced the donor pool by excluding the most influential country or region from the synthetic control unit.

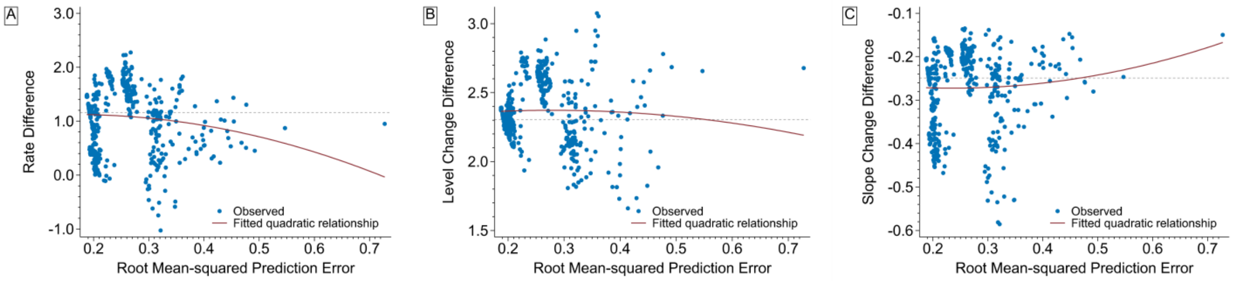

Figure S4 Scatterplot and fitted quadratic relationship between RMSPE and the estimated value of rate difference(A), level change(B) and slope change difference(C) from simulation analysis of the relationship between the composition of the synthetic control and the effect of TCP on the rate of natural increase.

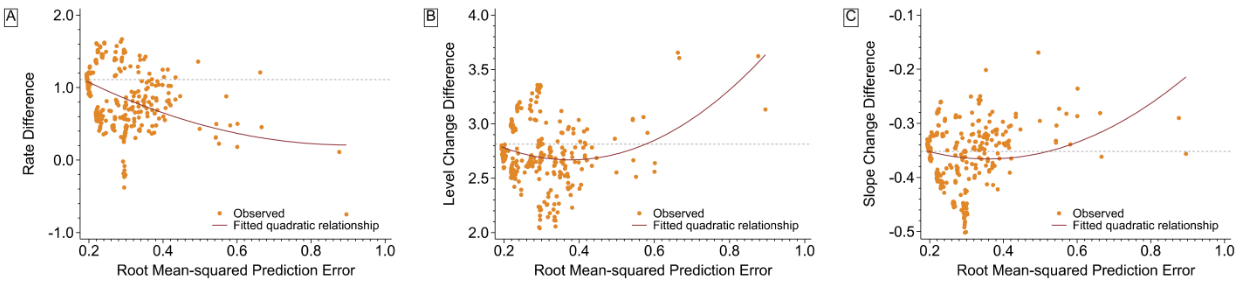

Figure S5 Scatterplot and fitted quadratic relationship between RMSPE and the estimated value of rate difference(A), level change(B) and slope change difference(C) from simulation analysis of the relationship between the composition of the synthetic control and the effect of TCP on the birth rate.
